# Structure‐based computational design of antibody mimetics: challenges and perspectives

**DOI:** 10.1002/2211-5463.13855

**Published:** 2024-06-26

**Authors:** Elton J. F. Chaves, Danilo F. Coêlho, Carlos H. B. Cruz, Emerson G. Moreira, Júlio C. M. Simões, Manassés J. Nascimento‐Filho, Roberto D. Lins

**Affiliations:** ^1^ Aggeu Magalhães Institute Oswaldo Cruz Foundation Recife Brazil; ^2^ Department of Fundamental Chemistry Federal University of Pernambuco Recife Brazil; ^3^ Institute of Structural and Molecular Biology University College London UK; ^4^ Fiocruz Genomics Network Brazil

**Keywords:** *de novo* design, deep learning, machine learning, protein engineering, protein structure

## Abstract

The design of antibody mimetics holds great promise for revolutionizing therapeutic interventions by offering alternatives to conventional antibody therapies. Structure‐based computational approaches have emerged as indispensable tools in the rational design of those molecules, enabling the precise manipulation of their structural and functional properties. This review covers the main classes of designed antigen‐binding motifs, as well as alternative strategies to develop tailored ones. We discuss the intricacies of different computational protein–protein interaction design strategies, showcased by selected successful cases in the literature. Subsequently, we explore the latest advancements in the computational techniques including the integration of machine and deep learning methodologies into the design framework, which has led to an augmented design pipeline. Finally, we verse onto the current challenges that stand in the way between high‐throughput computer design of antibody mimetics and experimental realization, offering a forward‐looking perspective into the field and the promises it holds to biotechnology.

## Graphical abstract

Computer graphics representation of an artificial intelligence–driven protein design pipeline, where building blocks are fed into the birth of a novel protein structure.

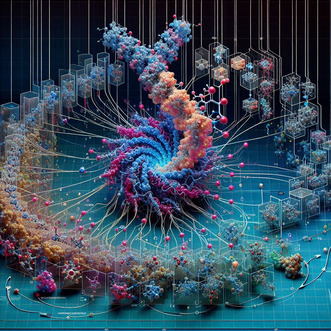

**Abbreviations**
∆∆G: binding free energy difference
AF2: AlphaFold2
AF3: AlphaFold3
AI: artificial intelligence
ANN: artificial neural network
dArmRP: designed armadillo repeat proteins
DARPIN: designed ankiryn repeat proteins
DL: deep learning
DPPM: denoising diffusion probability model
FN3: fibronecting type‐III
GN: generative model
hACE2: human angiotensin‐converting enzyme 2
IL‐17A: interleukin‐17A
kDa: kilodalton
mAb: monoclonal antibody
MC: Monte Carlo
ML: machine learning
MSA: multiple sequence alignment
MSE: mean square error
NMR: nuclear magnetic resonance
PDB: protein databank
R&D: research and development
RBD: receptor binding domain
RIF: rotamer interaction field
RMSD: root mean square deviation
SARS‐CoV‐2: severe acute respiratory syndrome coronavirus 2
SASA: solvent accessible surface area
VEGF‐A: vascular endothelial growth factor A
VHH: variable heavy domain

Recent advancements in therapeutic antibody research have led to significant progress in both key technologies and theoretical innovations. This encompasses the development of antibody‐drug conjugates, antibody‐conjugated nucleotides, bispecific antibodies, nanobodies, and various other antibody derivatives. Furthermore, therapeutic antibodies have been effectively combined with technologies from other fields, giving rise to novel interdisciplinary applications, including cell‐based therapies [1]. In fact, the biopharmaceutical industry is one of the most dynamic innovation and business ecosystems, with an estimated investment of hundreds of billions of dollars annually. In the United States alone, it accounted for 17% of dollars spent on domestic research and development (R&D) in the year of 2020, nearly doubling the investment on software development in the country [2]. Its main product, monoclonal antibodies (mAbs), can be designed to specifically target disease‐causing molecules or cells, minimizing off‐target effects. According to a report from Future Market Insights, the antibody therapy market in 2023 accounted for USD 235 billion and it is expected to reach USD 824 billion in the next decade. Most mAbs come from natural sources, offering biocompatibility advantage, and reducing the risk of adverse reactions when employed *in vivo*. They have been developed to treat a wide range of diseases, including cancer, autoimmune disorders, and infectious diseases. However, producing mAbs requires complex and highly specialized protein production technology, and its cost precludes population‐wide use of this class of molecules.

The development of synthetic antibody‐mimetics (proteins structurally not related to antibodies, but capable of exerting similar function) has been explored as an alternative to the limitations above. Unlike biopharmaceuticals, antibody mimetics offer simpler and scalable production *via* chemical synthesis or microbial fermentation. However, the development of an antibody mimetic typically required a significant investment in R&D. In addition to designing and optimizing novel structures (e.g., design target properties, engineer stability, solubility, and improve biocompatibility), validating their efficacy and safety *in vivo* may be challenging due to their novel and engineered nature, requiring extensive preclinical and clinical testing. Nevertheless, its versatility potential and cost of production are unmatched. A comparison of the main advantages and disadvantages of using antibody mimetics and conventional antibodies is summarized in Table 1. To date, nearly two‐dozen scaffold classes of antibody‐mimetics have been explored, in addition to a few tailored designs. Figure 1 illustrates the structure of the current most used scaffold classes, highlighting their binding domains. (As the focus of these review is on the computational design approaches for antibody mimetics, we suggest the review by Yu and colleagues for a more in‐depth biomedical applications for these molecules) [3].

**Table 1:** Comparison of the main advantages and disadvantages between antibody mimetics and conventional antibodies.

	Main advantages	Main disadvantages
Antibody mimetics	Size control: typically, smaller and simpler in structure compared to conventional antibodies, which allows for better tissue penetration and potentially reduced immunogenicity	Achieving high affinity: compared to conventional antibodies, achieving binding affinities and target specificity is challenging, often requiring multiple rounds of design
	Engineering flexibility: mimetics can be engineered with specific properties tailored to their intended applications (e.g., enhanced stability and/or binding affinity)	Clinical validation: extensive validation and clinical track record are needed, compared to conventional antibodies, potentially raising efficacy and safety concerns
	Versatility of administration: mimetics can be designed to control the *via* of administration. They are often small enough to be orally administered, if desired, offering advantages in terms of patient convenience and compliance	Shorter half‐life: mimetics may have shorter half‐lives in circulation compared to conventional antibodies, requiring either more frequent dosing for therapeutic applications or fusing to other proteins to enhance their half‐life
	Structural diversity: mimetics can be derived from various sources, including synthetic peptides, small proteins, or non‐protein molecules, and even novel scaffolds, providing a wide range of options for development	High development cost: development of mimetics by experimental means involves great financial risk, historically being mostly undertaken by the private sector
	Production cost: they can be engineered to be produced in prokaryotes and to yield large quantities	Lack of effector function: mimetics do not carry the antibody constant fraction region
Conventional antibodies	High specificity: conventional antibodies, particularly monoclonal antibodies, exhibit high specificity for their target antigens, which minimizes off‐target effects	Complex structure: conventional antibodies have a complex structure, making them expensive and challenging to produce at scale
	Natural recognition: they rely on the natural immune system's mechanisms for target recognition, ensuring biocompatibility	Immunogenicity: antibodies derived from non‐human sources can provoke immune responses, potentially limiting their therapeutic use
	Versatility: antibodies can be modified and engineered for various applications, including therapeutics, diagnostics, and research tools	Limited tissue penetration: their large size can hinder tissue penetration, affecting efficacy in certain therapeutic applications
	Long half‐life: IgG antibodies have a relatively long half‐life in bloodstream, providing sustained therapeutic effects	Storage and stability: antibodies require specific storage conditions and can degrade over time, affecting their shelf life and efficacy
	Well‐established production: large‐scale production methods for conventional antibodies are well‐established, facilitating manufacturing for commercial purposes	Production cost: it requires eukaryotic cell lines for production due to post‐translational modifications

**Fig. 1:**
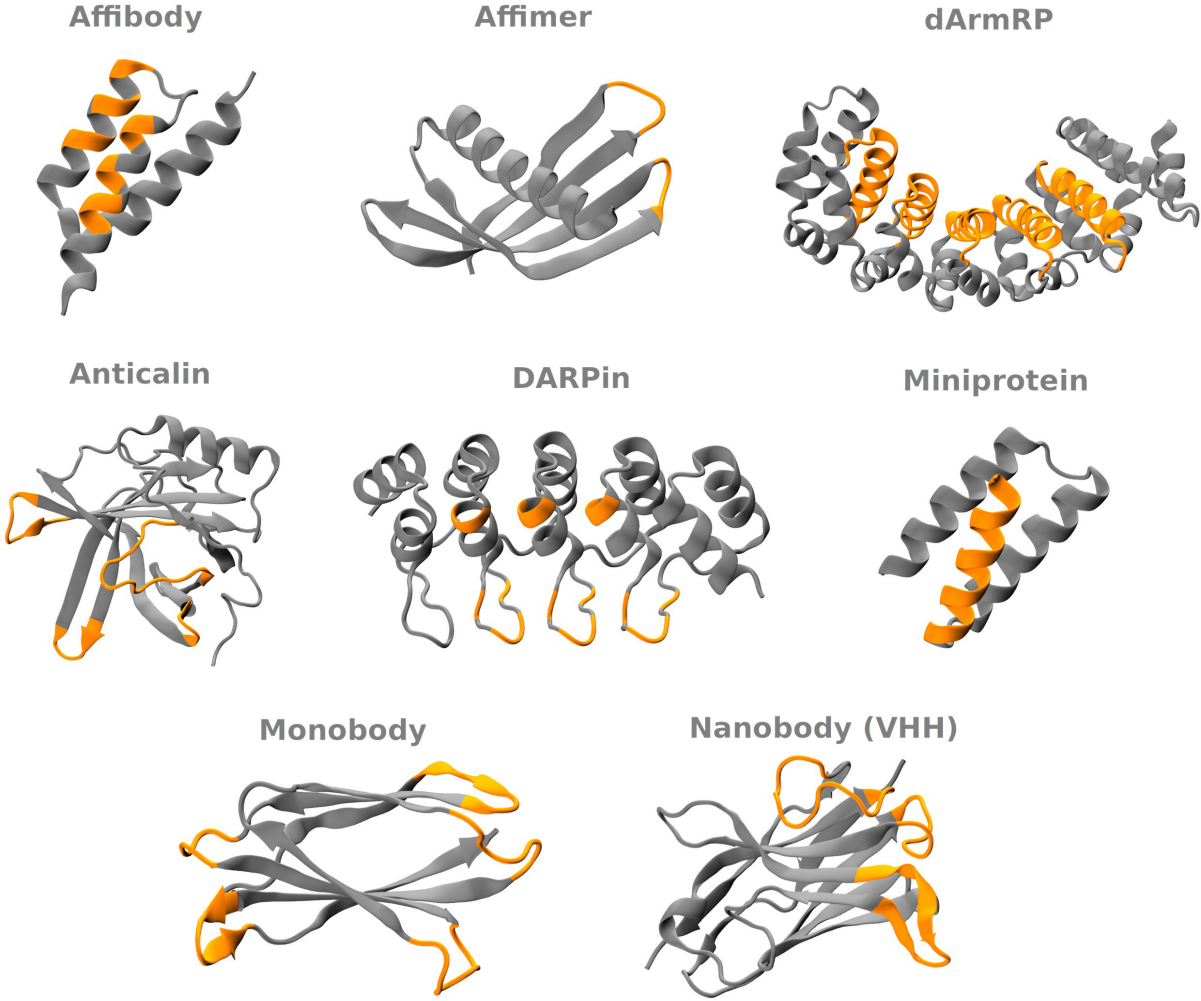
Protein structure scaffolds of the main antibody mimetics. Structures are shown in cartoon model, where the framework and binding domains are represented in gray and orange, respectively. The accession codes for each structure in the PDB and the amino acids on the binding domain are the following: 8DA4 (Affibody), residues 9–11, 13, 14, 17, 18, 24, 25, 27, 28, 31, and 32; 4N6T (Affimer), residues 60–71, and 98–100; 5AEI (dArmRP), residues 65–83, 108–126, 149–168, 192–210, 233–252, 91–101, 115–121, and 141–156; 1N0S (Anticalin), residues 31–43, 62–65, 90–93, and 117–123; 2XEE (DARPin), residues 43, 45, 46, 48, 56, 57; 7S5B (Mini‐protein), residues 1–16; 1TEN (Monobody), residues 813–818, 827–831, 840–846, 862–867, and 877–882; 1I3V (Nanobody), residues 26–32, 52–62, and 105–116.

As the field of computational protein design developed, these techniques have been employed as a way of speeding up the achievement of suitable structural properties (such as those mentioned above), thus reducing R&D‐related costs, especially those associated to the early stages of development. These methods have been mainly used to leverage the potential of already validated classes of antibody mimetics by harnessing, *in silico*, all possible sequences that fit the desired function criteria. However, the recent association of AI technology to computational protein design now allows the development of novel binders that do not rely on predetermined protein templates. It unfolds an unprecedent potential for exploration of the flourishing field beyond nature's protein portfolio.

## Main classes of antibody mimetics

### Affibody

Based on the B‐domain of staphylococcal protein A, it has a molecular weight of ca. 6 kDa. Affibodies are designed to bind to specific target molecules, such as proteins or peptides, with high affinity and specificity. Advantages over conventional antibodies include smaller size, simpler structure, and ease of engineering. Affibodies have applications in various areas including diagnostics, imaging, drug delivery, and targeted therapy [4]. Izokibep, an affibody‐based biopharmaceutical, was shown to bind to and inhibit the activity of IL‐17A in *in vitro* and *in vivo* assays using a murine model. It was also found to be safe and well‐tolerated in phase I and II clinical studies for the treatment of psoriatic arthritis [5].

### Affimer

Previously known as Adhiron, its scaffold is based on the human protease inhibitor, Stefin A [6]. Affimers have a molecular weight of ca. 11 kDa, and their structure contains four β‐sheets, one α‐helix, and two variable loops. These loops consist of nine amino acids each, used to design binding interfaces for a desired target. Up to date, affimers have been mainly designed by molecular or directed evolution techniques, where a diverse library of potential binding proteins is created and screened for those with the desired properties. They have been used as molecular probes for studying protein interactions, as diagnostic tools for detecting biomarkers or pathogens, and as therapeutic agents for targeting specific molecules involved in diseases such as cancer or inflammatory disorders [6, 7].

### dArmRP

Designed Armadillo Repeat Proteins (dArmRPs) have an armadillo domain, consisting of sequential armadillo repeats (8–12 internal repeats), each containing approximately 42 amino acids. They vary in molecular weight by 39 and 58 kDa. Each repeat consists of three α‐helices, designated as H1, H2, and H3. Inserted between the N and C terminus, the helical repeats protect the hydrophobic core from exposure to the solvent. They have been computationally designed to recognize and bind to peptide ligands, overcoming the limitation of antibody specificity upon peptide flexibility. Other uses include drug delivery, and molecular imaging [8, 9, 10].

### Anticalin

Derived from lipocalins, a family of naturally occurring proteins that typically bind to small hydrophobic molecules, anticalins were created through protein engineering techniques to have binding sites tailored for specific targets, such as drugs, metabolites, or other molecules of interest. Their structure consists of a cup‐shaped pocket weighing about 20 kDa. Biomedical applications include the delivery of biopharmaceuticals across the blood–brain barrier [11], and theranostic applications [12].

### DARPIN

Proteins composed by 33 amino acids ankyrin repeat motifs, arranged into two linked α‐helices in opposite directions, and connected to the subsequent repeat through an elongated β‐turn. DARPins are typically synthesized through combinatorial protein design using libraries comprising two to three ARPs repeated motifs. These motifs are sandwiched between positively and negatively charged N‐ and C‐terminal caps, typically incorporating six random positions within the β‐turn and the first α‐helix of each repeat. As the number of monomers can vary, DARPINs will have a minimum molecular weight of 14 kDa. The scaffold has been designed for several applications, such as antivirals [13, 14, 15], and cancer treatment. The most progressed DARPin compound in clinical development is Abicipar pegol, an antagonist of VEGF‐A. It is currently undergoing phase III trial to explore its potential to treat ophthalmic conditions including neovascular age‐related macular degeneration and diabetic macular edema [16].

### Miniprotein

This is a case of a tailored *de novo* design conceptualized by the group of David Baker [17, 18]. These small proteins are typically formed by fewer than 50 residues. Despite their small size, they can fold into three‐dimensional structures, often 3‐ or 4 helix bundles, with a molecular weight ranging from 5 to 10 kDa. As showcase, the Baker Labs developed designs against the RBD (receptor binding domain of spike protein) of SARS‐CoV‐2, with affinities ranging from 100 pm to 10 nm, and able to block virus infection *in vitro* [19].

### Monobody

Based on the human fibronectin type III domain (FN3), this scaffold has an immunoglobulin‐like fold, with a molecular weight ca. 10–15 kDa. Monobodies lack disulfide bonds, and thus, they are particularly suited as genetically encoded reagents to be used intracellularly [20], while the small and simple structure of monomeric monobodies confers increased tissue distribution. When designed in a bead‐on‐a‐string‐like assembly, multiple domains of FN3 can bind to different targets, overcoming the multi‐specificity challenge of conventional antibodies. Furthermore, full‐length fibronectin can fold into multiple conformations as part of its natural function, providing structural and sequence versatility to monobodies [21], with affinity and specificity that rival those of antibodies [22, 23].

### Nanobody

Nanobodies, also known as VHH or single‐domain antibodies, are a class of antibody fragments derived from the variable region of heavy‐chain antibodies found in camelids, such as camels, llamas, and alpacas. The proteins consist of a single monomeric antibody domain. With a molecular weight around 12–15 kDa, it makes them one‐tenth the size of conventional antibodies. Despite their small size, nanobodies retain high specificity and affinity for their target antigens, making them valuable tools in various biomedical applications. While they are generally obtained by animal immunization or phage display techniques, computer design of nanobodies have recently become popular [24]. *In silico* affinity maturation has also been used to improve the thermal stability and binding affinity of natural nanobodies [25].

## Computational protein design as the foundation for antibody mimetic development

The design of antibody mimetics is based on protein engineering principles. Our understanding of protein structure and function has matured significantly since the groundwork of Linus Pauling and Francis Crick in 1950s, allowing us to design proteins with specific properties. Protein engineering techniques can be classified as empiricism‐based (e.g., directed evolution, phage display) or mechanism‐based (e.g., comparative modeling, *de novo* design). Although purely experimental designs have been largely successful, they are cost and labor‐consuming, and the lessons learned are usually not applicable to unrelated systems [26]. In addition, evolution explores limited protein sequence space, leading to clustered natural protein families [27]. *De novo* design allows exploration of broader sequence space, leveraging protein biophysics principles.

Computational protein design methods are based on thermodynamics principles and biological observations, and they can be used for practically all classes of proteins [27, 28]. However, these methods rely on Anfinsen's Thermodynamic Hypothesis, that is, proteins fold into the lowest energy states that are accessible to their amino acid sequences [29]. In the last decade, rational design based on computational modeling and structural analysis emerged as a powerful strategy to engineer proteins with enhanced stability, activity, and/or specificity. It is based on the assumption that the geometry of a protein, together with the specific presentation of charges and molecular groups on its surface, determine its function. A given sequence of amino acids (primary structure) often leads to a specific three‐dimensional structure. On the other hand, different combinations of residues with similar properties can also lead to the same final topological structure. Protein design aims to determine an amino acid sequence that will fold into a three‐dimensional structure to perform a specific function. Thus, the key points are the configuration sampling method and the energy function used to predict stability and binding for searching the lowest energy model.

The advancement in rational protein design has historically been related to the progress of structure prediction methods and the increase in the number of experimentally determined target structures. Until three decades ago, most of computational techniques were limited to modifications through site‐directed mutagenic assays at binding sites, surfaces, and interfaces of well‐defined and untouched frameworks. This scenario has changed since 2003 after design of a novel protein from scratch called Top7. Using Monte Carlo search with molecular force fields and scoring functions, a novel protein was designed with unprecedented topology at the time [30]. To achieve the final model, several sequence design iterative cycles and backbone optimizations were performed. X‐ray crystallography and NMR resolved the structure of the unnatural 93‐mer α/β fold protein. Comparison with the model showed a backbone RMSD of approximately 1 Å, and the protein also exhibited remarkable thermodynamic stability [30]. That provided Top7 with a rich work portfolio that has vast implications on the most diverse areas of medicine and biotechnology as an ultra‐stable scaffold [31, 32]. Since then, structure‐based protein design has been marked by exceptional advances and significant increase in the number of proteins designed with high levels of complexity. However, we are just now experiencing a further substantial advance in the field. The recent use of neural networks on computational protein prediction (e.g., AlphaFold2 [33] and RoseTTAFold [34]) have allowed solving large protein structures with atomic precision and remarkable rapidness, overcoming half a century of challenges. These advances have paved ways for the development of robust, yet efficient, sampling algorithms, and sophisticate design methods.

## Design of antibody mimetics

**Fig. 2:**
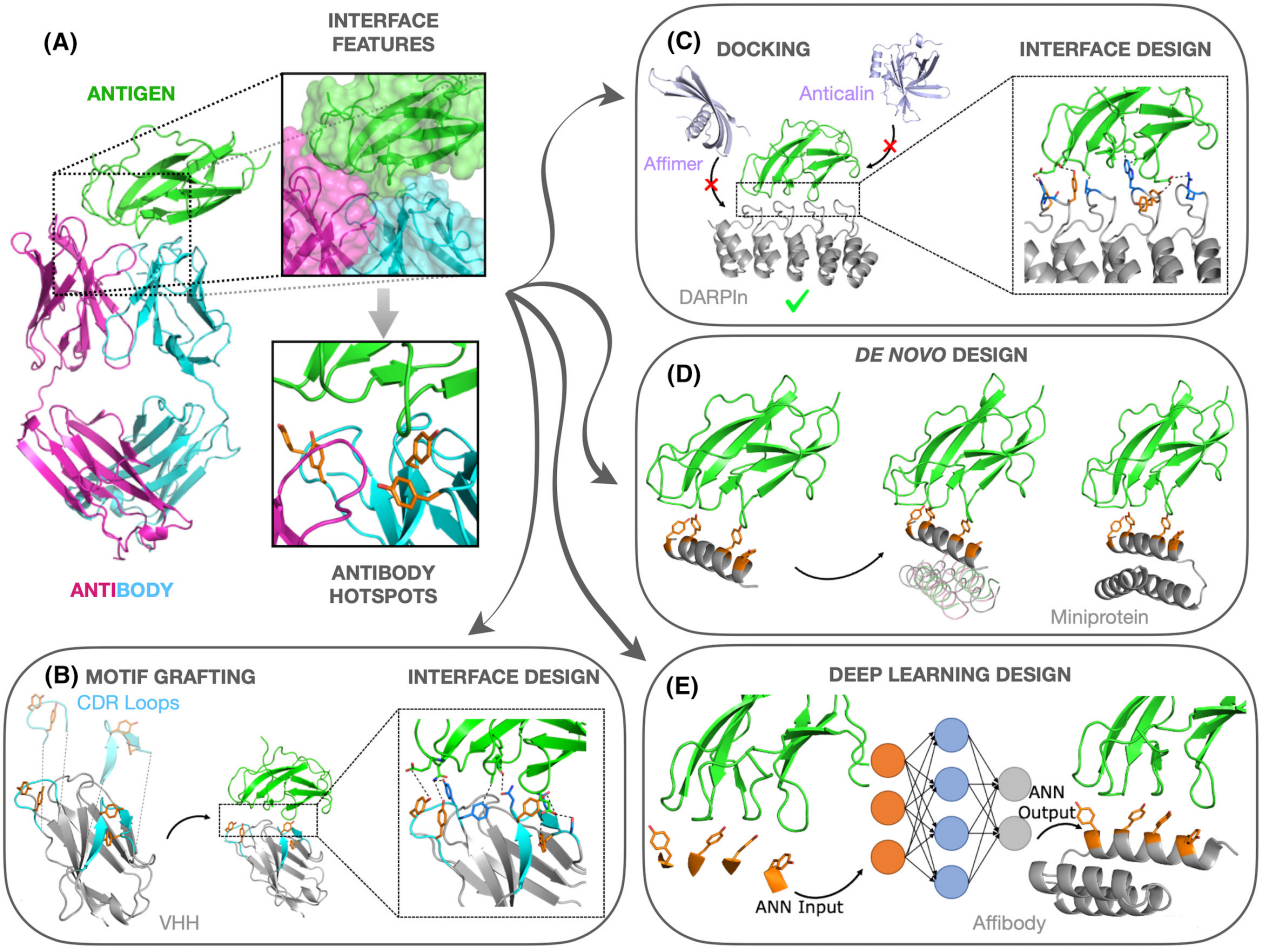
Computational workflow strategies for the design of antibody mimetics. (A) Initial step consists of obtaining the structural and physicochemical characteristics of the antigen target interface (or antigen–antibody interface), and if possible, to identify key‐residues for recognition. This information will be selectively passed on the design strategy used. (B) The Motif Grafting method requires structural data of antigen–antibody interface. Selected regions of the paratope that are considered important to recognition will be attempted to be grafted onto a variety of scaffolds. A scaffold that can withstand grafting will have its interface and topology sequence optimized to confer the desired affinity and protein stability, respectively. This example shows the grafting of CDR from conventional antibodies onto a VHH. (C) The Docking & Design method requires structural information from the antigen only. Scaffolds from a pre‐triaged structural library will be attempted to dock onto the antigen target surface, considering their shape complementarity. Once the two proteins are docked, mimetic will undergo interface optimization to achieve high affinity, and subsequently optimization of its scaffold (where the pre‐optimized interface sequence is kept fixed) to confer protein stability. It is exemplified here by attempting to dock the affimer, anticalin and DARPin mimetic classes. (D) *De Novo* Design creates a topology from scratch. It uses only structural data from the antigen only. However, a higher chance of success is achieved when antigen–antibody structural data is available. In the latter, a structural motif carrying the key‐interacting residues of the antibody is used as a starting point. Proteins are designed to vehicle the chosen motif, as close as possible from its native form. The example shows the building of a miniprotein. (E) As in the *De Novo* Design, one can use either structural data from the antigen only or antigen–antibody, if available. The example depicts the generation of an affibody using ANN, that uses as starting point selected key‐interacting residues from the antibody. Finally, it is worth noting that, even when key‐interacting residues from the antibody are used as starting point, they can be optimized (mutated) to generate a molecule with enhanced binding affinity (all presented examples are merely illustrative and therefore do not belong to any published study).

While protein prediction methods have matured in the last few years, protein complex prediction has lacked behind. Success is highly dependent on the strategy to estimate parameters that affect the binding affinity. Predicting association strength and complex structure accurately often requires combined techniques and experimental data guidance [35]. This is likely based on the electrostatic diversity of protein–protein interactions. Algorithms were initially designed to fold proteins, which almost invariably are formed by a hydrophobic core and a mostly hydrophilic solvent accessible area. In contrast, protein–protein interactions are seldom characterized by hydrophobic contacts only. Designing antibody‐mimetics considering target epitope molecular signature yields higher success rates than postdesign optimization. Based on that strategy, a number of target‐oriented computer protein design methods have been used to the development of antibody mimetics. The main techniques are discussed below, and a schematic workflow of each technique is shown in Fig. 2.

### Docking and design

When designing a new protein, sometimes the goal is just to modify positions in the amino acid sequence of an already known protein structure complex. In this case, the template is the native protein complex itself, and the design is performed on top of it. This method is commonly used to design proteins with improved binding affinities to other proteins or ligands [36]. Alternatively, key‐interacting residues in the antigen interface are selected as starting point for the design of the new protein (antibody mimetic). The idea is to search high‐resolution protein structures to be used as scaffolds with complementary shapes to antigens. Then, engineer interaction interfaces using local docking and designing steps. As in protein folding, protein association is driven by energy minimization, induced by van der Waals interactions, hydrophobic effect, electrostatic interaction, hydrogen bonding, shape, and chemical complementarity of the interaction partners [37]. Sequence optimization is performed every cycle, and a scoring function is used to find residues that stabilize the interaction and improve the binding propensity. Subsequently, the interaction interface sequence of the antibody mimetic remains fixed. Sequence optimization follows for remaining scaffold residues to ensure stability and solubility. A similar strategy to design interface is the so‐called hotspot‐centric approach, which consists of docking disembodied residues, selecting suitable scaffolds displaying residues at similar positions of the hotspots, and refining the interface [38, 39]. This approach has recently been used to design a biopharmaceutical targeting the conserved fusion loop region of the Envelope proteins of flaviviruses. The protein neutralized infections by Zika and Dengue serotype 1 and 2 viruses *in vitro*, with an EC_50_ on pair of human monoclonal antibodies [39]. Another alternative is the rotamer interaction field (RIF) docking, which searches for hotspots for protein interface interaction from scratch (*de novo*). Disembodied residue conformations are docked onto the target interface to create favorable hydrogen bonds and hydrophobic interactions [40]. Scaffolds that displays residues at similar positions are subsequently used as starting point for protein design. Residues onto the scaffold are mutated by the residues outputted by RIF docking, followed by interface and scaffold optimizations.

### Motif grafting design

Knowledge of the antigen–antibody interface structure is required as a starting point for the motif grafting method. Typically, antibody loops or selected regions of the interacting interface are grafted onto a protein scaffold [41]. The method consists of the following steps: define the motif to be transplanted, structurally align the motif to a putative carrier protein to determine the best region for grafting, transplant the motif, and redesign the scaffold protein around the grafted motif to ensure folding and stability of the chimeric protein. After the three‐dimensional alignment, one can choose between keeping the backbone scaffold and grafting only the side chains of the antibody (side‐chain grafting) or discarding the structural elements of the carrier, replacing it completely with the structural motif of the antibody (backbone‐grafting). A flexible‐backbone remodeling is employed to optimize the conformation of the protein skeleton after its modification [42]. Nevertheless, motif grafting by sidechain or backbone replacement faces limitations when the motif is too complex to find a structurally compatible protein scaffold. Proteins sporting a single immunoglobulin domain motif (e.g., VHH, selectins, ankyrin repeat proteins) are more commonly used with this technique.

### *De novo* design

Template‐based techniques have found several critical restrictions, imposed by strict adjustments of motifs onto scaffolds as well as the inconvenient requirement for well‐defined and suitable frameworks. Comparative methods rely on known homologous structures, limiting design to pre‐existing interfaces and excluding exploration of new sites [37]. To address the design of antibody mimetics to more complex epitopes, *de novo* methods represents a powerful alternative. It has been used to design new proteins around a given motif/epitope [43, 44].

*De novo* proteins began to be designed around binding motifs using minimal knowledge of their interactions, but without prior knowledge of scaffold atom‐positions. At first, these methods were considered *ab initio* or template‐free techniques, as they did not use any known structure as a base. As techniques have become more sophisticated, the boundaries between different categories have become less clear [45]. There are methods today that are considered hybrids between template‐free, and template‐based. In *de novo* design, the target sequence is not compared with known proteins but to a blueprint of α‐helix and β‐sheet fragments, creating low‐energy structures [27]. Folding is based on the basic principles of the diffusion‐collision model developed by Karplus and Weaver [46], where once a local thermodynamically favorable interaction is formed, it is maintained, creating a bias by bringing together other contacts into spatial proximity. The process is repeated until the complete folding of the protein. Generally, pipelines are conceived through three steps: (a) The core is built first by an assembling process starting from valine‐based secondary structures in the presence of the target; (b) the sequence is subsequently tailored on fixed backbone, taking into account the chemical environment (which also includes the antigen interface) and amino acid occurrence in secondary structure elements through layer‐based design approaches; and (c) the sampling of possible backbone conformations is done using a library of short amino acid fragments based on the distribution of homologous structures taken from the PDB [45]. The fragment library covers accessible local structures, while side chains are assembled from a rotameric library. The final structure is assembled using Metropolis/Monte Carlo [47, 48]. Binding affinity is achieved through successive interface design calculations at the interface with the aim of maximizing the number of buried hydrogen‐bonds and creating hydrophobic contacts as good as commonly found in native protein–protein complexes [49]. Although binding free energy would be expected to be the most relevant feature, due to the empiricism nature of the scoring functions, other interface variables must be considered for a proper and more realistic interface description. Interfaces are evaluated by comparing the difference in binding free energies (∆∆G), solvent accessibility surface area (SASA), shape complementarity, number of total and unsaturated hydrogen bonds, and ∆∆G/SASA of each decoy to the pool of generated structures. Although these energetic quantities do not find adherence in reality and therefore cannot be directly related to experimental measurements, they are crucial to triage the best candidate out of several thousand, or even a few million, generated designs. We highlight as showcase a work from the Baker's group that have recently harnessed the power of *de novo* methods to design a potent antibody mimetic against infection by the SARS‐CoV‐2 virus [19]. Miniproteins were developed to specifically block the interaction of human angiotensin‐converting enzyme 2 (hACE2) to the receptor binding domain (RBD) from SARS‐CoV‐2 Spike protein, and thereby preventing the entrance of the virus into host cells. They showed that two selected candidates, from several thousand designs, prevented virus entry into host cells, and prevented lung disease and pathology in mice [50].

### Machine and deep learning–driven design

Predicting binder‐target strengths with experimental precision is crucial for picking out the best candidates. Nevertheless, accurate calculation of the absolute binding free energies for protein–protein interactions has posed a challenge in protein design methods. Historically, calculation of protein–protein free energies at experimental accuracy were restricted to enhanced sampling methods, which are highly demanding in terms of computational requirements. The advent of artificial intelligence (AI) in the most diverse areas of knowledge has inspired the development of new approaches with great potential for engineering and triage protein–protein complexes without prior structural knowledge, at exceptional performance. In the last few years, the community has resourced from machine and deep learning algorithms to reweigh the molecular feature contributions to experimental binding free energies. The approach has allowed for similar accuracy to more costly methods, at an unprecedent efficiency, making it possible to triage high‐affinity binders, from thousands or even millions of candidates, in a high‐throughput fashion [51, 52, 53]. In addition, AI has also been used to design antibody mimetic scaffolds. It provides advantage over template‐based and *ab initio* methods. The former cannot predict new protein folds. While the latter may provide a greater range of new topology possibilities, it requires extensive sampling that results in a higher computational cost.

AI refers to systems or machines that imitate human intelligence. More specifically, machine learning (ML) and deep learning (DL) are subsets of AI that focus on building or improving predictive models that learn from data or identify informative groupings within data. ML has been used to improve existing antibodies, but most developed algorithms rely on deep sequencing or deep mutational scans for training data, and a shortcoming of these methods is that they are often specific classes of antibodies and applying them to other antibodies would require training with new data [54]. Few examples have been able to generate new antibodies and antigens without antibody‐specific sequencing data [55, 56, 57].

Among AI methods, artificial neural network (ANN) and generative models (GM) have gained a lot of attention in the development of methods for structural biology, whether in the description of interactions between biomolecules or for structure modeling [58, 59, 60, 61, 62]. Neural networks, a set of DL techniques, are mathematical models that mimic the connectivity and behavior of neurons in the brain. Artificial neurons, which are the building blocks of ANNs, are simply mathematical functions that convert inputs to outputs in a specific way. To create an ANN, artificial neurons are organized into layers, with the output of one layer being the input of the next. On the other hand, GM is designed to generate new data instances that resemble the training data they were trained on. They learn the underlying structure of the data and then use this knowledge to generate new samples. Among the ANN and GM models available in the literature for protein modeling, two stand out: AlphaFold2 [33] and RFdiffusion [34].

AlphaFold2 (AF2) predicts protein structures by leveraging neural networks and training procedures based on evolutionary, physical, and geometric constraints [33]. The core of AF2's architecture is the Evoformer block, understanding how different parts of the protein are related to each other in 3D space and represents the protein as a graph where each amino acid is a node and the connections between them are edges. Evoformer uses two input representations: the Pair Representation and the Multiple Sequence Alignment (MSA). The former captures the relationships between pairs of amino acids in a matrix describing how two amino acids interact to each other, while the MSA matrix represents homologous sequences, identifying positions prone to simultaneous mutations (coevolution) and potential contact points. Evoformer updates these representations through 48 blocks, ensuring accurate 3D structure representation by applying geometric constraints and using axial attention to focus on critical information. This continuous information exchange between the MSA and pair representations enables precise structure predictions. AF2 achieved top performance in CASP14 with a median backbone accuracy of 0.96 Å RMSD and an all‐atom accuracy of 1.5 Å RMSD [33]. AF2 was also adapted to predict multichain complexes (AlphaFold‐Multimer) [63] and benchmarked for antibody–antigen interactions, achieving 43% top‐ranked results for various protein complexes [64]. Recently, AlphaFold3 (AF3) was released [65], featuring a diffusion‐based architecture capable of predicting structures containing proteins, ions, nucleic acids, and small molecules, with improved accuracy for antibody–antigen complexes. AF3 replaces Evoformer with a simpler module and predicts atom coordinates using a diffusion module. The MSA is processed in four blocks and incorporated into the pairwise representation, which is then processed through 48 blocks in the new Pairformer module. Following, the diffusion module refines the initial atom coordinates through a denoising process. AF3 surpasses classical docking tools and shows enhanced accuracy for protein complexes, including antibody‐protein interactions [65].

The RFdiffusion method replaces the physical‐based Rosetta methods to DL approaches, aiming to predict diverse proteins and scaffold‐free binder interactions with atomic accuracy and unprecedented success [34]. RFdiffusion is an updated version of RoseTTAFold [66] that uses denoising diffusion probability models (DPPMs) to generate low‐resolution backbone models, and then use the ProteinMPNN network [58] to subsequently design sequences encoding these structures. Binders are designed similarly to creating photo‐realistic images from textual instructions, resulting in novel proteins with higher binding potential and experimental success. The denoising process acts on a random sample of residue backbone, through an interactive DL‐based design workflow, which disrupts coordinates toward true proteins, by minimizing the mean square error (MSE) to design the sequences. The authors demonstrated that RFDifusion is capable of generating binders for proteins used as target context, by selecting input residues in the target chain (defined as hotspots) to which the designed chain binds. The proof of concept was carried out with the target proteins Hemagglutinin Influenza A H1, Interleukin‐7‐α Receptor, Programmed Death Ligand 1, Insulin Receptor and Tropomyosin Kinase A Receptor, showing the potential of RFdiffusion for binders designing [34].

More recently, a new generative AI model was released to the public for use in structural biology. The 310 CoPILOT model was developed by 310 AI as an AI Chat for Designer Bio (https://310.ai/). This model allows you to perform tasks in structural biology through a web‐based chat platform, making use of third‐party tools such as search for and load proteins from the UniProt database, compare proteins using the TM‐Align method [67], fold proteins using the ESM Fold method [68], and design with ProteinMPNN model [58]. Furthermore, it also allows the use of 310.ai's own algorithm for designing new proteins. For binder design, the model allows the user to fold an antibody mimetic and then dock it with the target structure. Up to date, it is a tool still under development that requires a considerable amount of work to deliver its promises.

## Challenges and perspectives

The recent progress on the computer engineering of antibody mimetics has eased, but not eliminated the challenges that stand between direct design and experimental application. Successful design of antibody mimetics often requires several rounds of experimental validation. Through this process, it is possible to identify the required changes in the design or computational protocol to fine tune the structural changes needed to achieve its desired biological function [45].

In computational protein design, the quality of the final model depends on the efficiency of sampling and on the accuracy of the energy function [28]. Commonly, the configuration sampling method is a time‐independent stochastic process, such as Monte Carlo (MC) [69]. However, the stochastic nature of the method leads to not sampling every regional energy minima [70]. The free energy of binding, which is directly related to the binding affinity, is the most important indicator of a protein‐binding strength, and the most challenging to predict. The energy function is usually based on classical molecular mechanics force‐fields associated with other empirical terms that employ simplified interaction potentials, offering computational speed but at the expense of a certain degree of accuracy [27, 70]. Another well‐known limitation for computational protein design is the solvent treatment. Water molecules are important for the structure, stability, dynamics, and function of proteins [71] and generally should not be treated as an implicit interaction agent due to importance of desolvation penalty. The most frequent reasons for failure in protein design are insolubility and the formation of unintended oligomeric states. Proteins that bind to other proteins usually have hydrophobic residues on their surface, which may lead to unanticipated intermolecular hydrophobic interactions and aggregation. Increasing the robustness of designs will require improvements in the accuracy of the energy function not only for free energy binding, but also thermodynamic stability of monomeric proteins [27]. In addition, for such calculations to be useful in protein–protein binding discovery, where it is common to produce *in silico* millions of candidates, the predictions must be rapidly computed, preferably within at most a few hours, and they should also be accurate and reproducible.

In the last few years, AI methods have found use in all aspects of protein design, from weighting scoring functions and sampling space enhancement to the design process itself. These models can achieve high accuracy when predicting binding free energies. However, this accuracy is highly dependent on the quality of the experimental data used for training [72, 73]. In addition, while predicting binding free energies is key to design antibody mimetics, other aspects of developability need to be addressed, which include selectivity, stability, aggregation prevention, solubility, biocompatibility, deimmunization, bioavailability, and clearance rate. Other challenges that cannot be predicted, so far, include some proteins that are expressed recombinantly might be the toxic for the prokaryotic cell line used or pose other complexities of the bacterium's biology [27]. Therefore, unless these issues are met, the process of protein validation and progression to preclinical and clinical studies will still be largely hindered.

On the experimental side, current attempts to reduce the time required to validate a protein's usefulness in clinical settings include the implementation of automated processes aimed at promoting scalability of testing in a compatible time frame. Successful examples have shown that fully automated laboratories (also known as self‐driving labs) can achieve higher standards of accuracy and quality controls in *in vitro* assays to determine protein biocompatibility and function, when compared to their conventional counterparts [74, 75]. Although automation offers a glimpse of hope toward accelerating the translatability of computationally designed antibody mimetics, this approach is hardly a tangible solution for the vast majority of research groups due to its inherent high cost, need for stringent cybersecurity and the intrinsic characteristics of a molecular biology laboratory [74].

Considering the current issues, the efficient translation from computational design of synthetic antibody mimetics to real‐world applications seem to lie on the comprehensive implementation of AI methods into the design framework so that it goes beyond achieving binding affinity and stability. Toward this end, we envision that a multiple context optimization engine that combines protein structure, protein language, protein images, and biological labels may prove crucial at designing the next generation of novel antibody mimetics.

## Conflict of interest

The authors declare no conflict of interest.

## Author contributions

EJFC, EGM, JCMS, and MJN performed an in‐depth review of antibody mimetics; DFC and CHBC have reviewed the computational methodologies to design antibody mimetics. RDL has conceptualized the manuscript, oversaw the work, and wrote the final version. All coauthors have approved the submission of this manuscript.

## References

[1] Wang Z , Wang G , Lu H , Li H , Tang M and Tong A (2022) Development of therapeutic antibodies for the treatment of diseases. Mol Biomed 3, 35. 36418786 10.1186/s43556-022-00100-4 PMC9684400

[2] America, P RaMo (2020) Biopharmaceuticals in perspective.

[3] Yu X , Yang YP , Dikici E , Deo SK and Daunert S (2017) Beyond antibodies as binding partners: the role of antibody mimetics in bioanalysis. Annu Rev Anal Chem (Palo Alto Calif) 10, 293–320. 28375702 10.1146/annurev-anchem-061516-045205 PMC5895458

[4] Du W , Jiang P , Li Q , Wen H , Zheng M , Zhang J , Guo Y , Yang J , Feng W , Ye S *et al*. (2023) Novel affibody molecules specifically bind to SARS‐CoV‐2 spike protein and efficiently neutralize delta and omicron variants. Microbiol Spectr 11, e0356222. 36511681 10.1128/spectrum.03562-22 PMC9927262

[5] Klint S , Feldwisch J , Gudmundsdotter L , Dillner Bergstedt K , Gunneriusson E , Höidén Guthenberg I , Wennborg A , Nyborg AC , Kamboj AP , Peloso PM *et al*. (2023) Izokibep: preclinical development and first‐in‐human study of a novel IL‐17A neutralizing affibody molecule in patients with plaque psoriasis. MAbs 15, 2209920. 37184136 10.1080/19420862.2023.2209920 PMC10187109

[6] Stadler LKJ , Hoffmann T , Tomlinson DC , Song Q , Lee T , Busby M , Nyathi Y , Gendra E , Tiede C , Flanagan K *et al*. (2011) Structure−function studies of an engineered scaffold protein derived from Stefin A. II: development and applications of the SQT variant. Protein Eng Des Sel 24, 751–763. 21616931 10.1093/protein/gzr019

[7] Ackermann M , Morse BA , Delventhal V , Carvajal IM and Konerding MA (2012) Anti‐VEGFR2 and anti‐IGF‐1R‐adnectins inhibit Ewing's sarcoma A673‐xenograft growth and normalize tumor vascular architecture. Angiogenesis 15, 685–695. 22914877 10.1007/s10456-012-9294-9

[8] Parmeggiani F , Pellarin R , Larsen AP , Varadamsetty G , Stumpp MT , Zerbe O , Caflisch A and Plückthun A (2008) Designed armadillo repeat proteins as general peptide‐binding scaffolds: consensus design and computational optimization of the hydrophobic core. J Mol Biol 376, 1282–1304. 18222472 10.1016/j.jmb.2007.12.014

[9] Alfarano P , Varadamsetty G , Ewald C , Parmeggiani F , Pellarin R , Zerbe O , Plückthun A and Caflisch A (2012) Optimization of designed armadillo repeat proteins by molecular dynamics simulations and NMR spectroscopy. Protein Sci 21, 1298–1314. 22767482 10.1002/pro.2117 PMC3631359

[10] Madhurantakam C , Varadamsetty G , Grütter MG , Plückthun A and Mittl PRE (2012) Structure‐based optimization of designed armadillo‐repeat proteins. Protein Sci 21, 1015–1028. 22544642 10.1002/pro.2085 PMC3403439

[11] Nästle L , Deuschle FC , Morath V and Skerra A (2023) FerryCalin: an engineered lipocalin protein directed against the transferrin receptor with potential for brain drug delivery. Chembiochem 24, e202200795. 37005222 10.1002/cbic.202200795

[12] Deuschle F‐C , Morath V , Schiefner A , Brandt C , Ballke S , Reder S , Steiger K , Schwaiger M , Weber W and Skerra A (2020) Development of a high affinity anticalin® directed against human CD98hc for theranostic applications. Theranostics 10, 2172–2187. 32089738 10.7150/thno.38968 PMC7019167

[13] Walser M , Mayor J and Rothenberger S (2022) Designed ankyrin repeat proteins: a new class of viral entry inhibitors. Viruses 14, 2242. 36298797 10.3390/v14102242 PMC9611651

[14] Rothenberger S , Hurdiss DL , Walser M , Malvezzi F , Mayor J , Ryter S , Moreno H , Liechti N , Bosshart A , Iss C *et al*. (2022) The trispecific DARPin ensovibep inhibits diverse SARS‐CoV‐2 variants. Nat Biotechnol 40, 1845–1854. 35864170 10.1038/s41587-022-01382-3 PMC9750863

[15] Stojcheva N , Gladman S , Soergel M , Zitt C , Drake R , Lockett T , Marchand C , Fustier P , Stavropoulou V , Fernandez E *et al*. (2023) Ensovibep, a SARS‐CoV‐2 antiviral designed ankyrin repeat protein, is safe and well tolerated in healthy volunteers: results of a first‐in‐human, ascending single‐dose phase 1 study. Br J Clin Pharmacol 89, 2295–2303. 37057679 10.1111/bcp.15747

[16] Smithwick E and Stewart MW (2017) Designed ankyrin repeat proteins: a look at their evolving use in medicine with a focus on the treatment of chorioretinal vascular disorders. Antiinflamm Antiallergy Agents Med Chem 16, 33–45. 28464780 10.2174/1871523016666170502115816

[17] Baker EG , Bartlett GJ , Porter Goff KL and Woolfson DN (2017) Miniprotein design: past, present, and prospects. Acc Chem Res 50, 2085–2092. 28832117 10.1021/acs.accounts.7b00186

[18] Ożga K and Berlicki Ł (2022) Design and engineering of miniproteins. ACS Bio Med Chem Au 2, 316–327. 10.1021/acsbiomedchemau.2c00008 PMC10125317 37102166

[19] Cao L , Goreshnik I , Coventry B , Case James B , Miller L , Kozodoy L , Chen Rita E , Carter L , Walls Alexandra C , Park Y‐J *et al*. (2020) De novo design of picomolar SARS‐CoV‐2 miniprotein inhibitors. Science 370, 426–431. 32907861 10.1126/science.abd9909 PMC7857403

[20] Hantschel O , Biancalana M and Koide S (2020) Monobodies as enabling tools for structural and mechanistic biology. Curr Opin Struct Biol 60, 167–174. 32145686 10.1016/j.sbi.2020.01.015 PMC7370805

[21] Chandler PG and Buckle AM (2020) Development and differentiation in monobodies based on the fibronectin type 3 domain. Cells 9, 610. 32143310 10.3390/cells9030610 PMC7140400

[22] Diem MD , Hyun L , Yi F , Hippensteel R , Kuhar E , Lowenstein C , Swift EJ , O'Neil KT and Jacobs SA (2014) Selection of high‐affinity centyrin FN3 domains from a simple library diversified at a combination of strand and loop positions. Protein Eng Des Sel 27, 419–429. 24786107 10.1093/protein/gzu016

[23] Sha F , Salzman G , Gupta A and Koide S (2017) Monobodies and other synthetic binding proteins for expanding protein science. Protein Sci 26, 910–924. 28249355 10.1002/pro.3148 PMC5405424

[24] Bai Z , Wang J , Li J , Yuan H , Wang P , Zhang M , Feng Y , Cao X , Cao X , Kang G *et al*. (2023) Design of nanobody‐based bispecific constructs by in silico affinity maturation and umbrella sampling simulations. Comput Struct Biotechnol J 21, 601–613. 36659922 10.1016/j.csbj.2022.12.021 PMC9822835

[25] Tam C , Kukimoto‐Niino M , Miyata‐Yabuki Y , Tsuda K , Mishima‐Tsumagari C , Ihara K , Inoue M , Yonemochi M , Hanada K , Matsumoto T *et al*. (2023) Targeting Ras‐binding domain of ELMO1 by computational nanobody design. Commun Biol 6, 284. 36932164 10.1038/s42003-023-04657-w PMC10023680

[26] Pantazes RJ , Grisewood MJ and Maranas CD (2011) Recent advances in computational protein design. Curr Opin Struct Biol 21, 467–472. 21600758 10.1016/j.sbi.2011.04.005

[27] Huang P‐S , Boyken SE and Baker D (2016) The coming of age of de novo protein design. Nature 537, 320. 27629638 10.1038/nature19946

[28] Gainza P , Nisonoff HM and Donald BR (2016) Algorithms for protein design. Curr Opin Struct Biol 39, 16–26. 27086078 10.1016/j.sbi.2016.03.006 PMC5065368

[29] Anfinsen CB (1973) Principles that govern the folding of protein chains. Science 181, 223. 4124164 10.1126/science.181.4096.223

[30] Kuhlman B , Dantas G , Ireton GC , Varani G , Stoddard BL and Baker D (2003) Design of a novel globular protein fold with atomic‐level accuracy. Science 302, 1364–1368. 14631033 10.1126/science.1089427

[31] Soares TA , Boschek CB , Apiyo D , Baird C and Straatsma TP (2010) Molecular basis of the structural stability of a Top7‐based scaffold at extreme pH and temperature conditions. J Mol Graph Model 28, 755–765. 20185346 10.1016/j.jmgm.2010.01.013

[32] Viana IFT , Soares TA , Lima LFO , Marques ETA , Krieger MA , Dhalia R and Lins RD (2013) De novo design of immunoreactive conformation‐specific HIV‐1 epitopes based on Top7 scaffold. RSC Adv 3, 11790–11800.

[33] Jumper J , Evans R , Pritzel A , Green T , Figurnov M , Ronneberger O , Tunyasuvunakool K , Bates R , Žídek A , Potapenko A *et al*. (2021) Highly accurate protein structure prediction with AlphaFold. Nature 596, 583–589. 34265844 10.1038/s41586-021-03819-2 PMC8371605

[34] Watson JL , Juergens D , Bennett NR , Trippe BL , Yim J , Eisenach HE , Ahern W , Borst AJ , Ragotte RJ , Milles LF *et al*. (2023) De novo design of protein structure and function with RFdiffusion. Nature 620, 1089–1100. 37433327 10.1038/s41586-023-06415-8 PMC10468394

[35] Drake ZC , Seffernick JT and Lindert S (2022) Protein complex prediction using Rosetta, AlphaFold, and mass spectrometry covalent labeling. Nat Commun 13, 7846. 36543826 10.1038/s41467-022-35593-8 PMC9772387

[36] Tinberg CE , Khare SD , Dou J , Doyle L , Nelson JW , Schena A , Jankowski W , Kalodimos CG , Johnsson K , Stoddard BL *et al*. (2013) Computational design of ligand‐binding proteins with high affinity and selectivity. Nature 501, 212–216. 24005320 10.1038/nature12443 PMC3898436

[37] Marchand A , Van Hall‐Beauvais AK and Correia BE (2022) Computational design of novel protein–protein interactions – an overview on methodological approaches and applications. Curr Opin Struct Biol 74, 102370. 35405427 10.1016/j.sbi.2022.102370

[38] Schreiber G and Fleishman SJ (2013) Computational design of protein–protein interactions. Curr Opin Struct Biol 23, 903–910. 23993666 10.1016/j.sbi.2013.08.003

[39] Viana IFT , Cruz CHB , Athayde D , Adan WCS , Xavier LSS , Archer M and Lins RD (2023) In vitro neutralisation of zika virus by an engineered protein targeting the viral envelope fusion loop. Mol Syst Design Eng 8, 516–526.

[40] Dou J , Vorobieva AA , Sheffler W , Doyle LA , Park H , Bick MJ , Mao B , Foight GW , Lee MY , Gagnon LA *et al*. (2018) De novo design of a fluorescence‐activating beta‐barrel. Nature 561, 485–491. 30209393 10.1038/s41586-018-0509-0 PMC6275156

[41] Silva DA , Correia BE and Procko E (2016) Motif‐driven design of protein‐protein interfaces. Methods Mol Biol 1414, 285–304. 27094298 10.1007/978-1-4939-3569-7_17

[42] Correia BE , Ban YE , Friend DJ , Ellingson K , Xu H , Boni E , Bradley‐Hewitt T , Bruhn‐Johannsen JF , Stamatatos L , Strong RK *et al*. (2011) Computational protein design using flexible backbone remodeling and resurfacing: case studies in structure‐based antigen design. J Mol Biol 405, 284–297. 20969873 10.1016/j.jmb.2010.09.061

[43] Bonet J , Wehrle S , Schriever K , Yang C , Billet A , Sesterhenn F , Scheck A , Sverrisson F , Veselkova B , Vollers S *et al*. (2018) Rosetta FunFolDes – a general framework for the computational design of functional proteins. PLoS Comput Biol 14, e1006623. 30452434 10.1371/journal.pcbi.1006623 PMC6277116

[44] Correia BE , Bates JT , Loomis RJ , Baneyx G , Carrico C , Jardine JG , Rupert P , Correnti C , Kalyuzhniy O , Vittal V *et al*. (2014) Proof of principle for epitope‐focused vaccine design. Nature 507, 201–206. 24499818 10.1038/nature12966 PMC4260937

[45] Kuhlman B and Bradley P (2019) Advances in protein structure prediction and design. Nat Rev Mol Cell Biol 20, 681–697. 31417196 10.1038/s41580-019-0163-x PMC7032036

[46] Karplus M and Weaver DL (1994) Protein folding dynamics: the diffusion‐collision model and experimental data. Protein Sci 3, 650–658. 8003983 10.1002/pro.5560030413 PMC2142854

[47] Rohl CA , Strauss CEM , Misura KMS and Baker D (2004) Protein structure prediction using Rosetta. Methods Enzymol 383, 66–93. 15063647 10.1016/S0076-6879(04)83004-0

[48] Simons KT , Kooperberg C , Huang E and Baker D (1997) Assembly of protein tertiary structures from fragments with similar local sequences using simulated annealing and bayesian scoring functions. J Mol Biol 268, 209–225. 9149153 10.1006/jmbi.1997.0959

[49] Cao L , Coventry B , Goreshnik I , Huang B , Sheffler W , Park JS , Jude KM , Marković I , Kadam RU , Verschueren KHG *et al*. (2022) Design of protein‐binding proteins from the target structure alone. Nature 605, 551–560. 35332283 10.1038/s41586-022-04654-9 PMC9117152

[50] Case JB , Chen RE , Cao L , Ying B , Winkler ES , Johnson M , Goreshnik I , Pham MN , Shrihari S , Kafai NM *et al*. (2021) Ultrapotent miniproteins targeting the SARS‐CoV‐2 receptor‐binding domain protect against infection and disease. Cell Host Microbe 29, 1151–1161.e5. 34192518 10.1016/j.chom.2021.06.008 PMC8221914

[51] Chaves E , Mhrous E , Nascimento‐Filho M , Cruz C , Ferraz M and Lins R (2023) Prediction of absolute protein–protein binding free energy by a super learner model. ChemRxiv. doi: 10.26434/chemrxiv-2023-zq1nj PMC11898044 39973292

[52] Ferraz MVF , Neto JCS , Lins RD and Teixeira ES (2023) An artificial neural network model to predict structure‐based protein–protein free energy of binding from Rosetta‐calculated properties. Phys Chem Chem Phys 25, 7257–7267. 36810523 10.1039/d2cp05644e

[53] Ferraz MVF , Viana IFT , Coêlho DF , da Cruz CHB , de Arruda Lima M , de Luna Aragão MA and Lins RD (2022) Association strength of E6 to E6AP/p53 complex correlates with HPV‐mediated oncogenesis risk. Biopolymers 113, e23524. 36281776 10.1002/bip.23524

[54] Notin P , Rollins N , Gal Y , Sander C and Marks D (2024) Machine learning for functional protein design. Nat Biotechnol 42, 216–228. 38361074 10.1038/s41587-024-02127-0 PMC13159571

[55] Varun RS , Theodora UJB , Brian LH and Peter SK (2023) Inverse folding of protein complexes with a structure‐informed language model enables unsupervised antibody evolution. bioRxiv. doi: 10.1101/2023.12.19.572475

[56] Amir S , Matt M , George K , Andrea KS , John MS , Edriss Y , Cailen M , Robel H , Richard S , Julian A *et al*. (2024) Unlocking *de novo* antibody design with generative artificial intelligence. bioRxiv. doi: 10.1101/2023.01.08.523187

[57] Hie BL , Shanker VR , Xu D , Bruun TUJ , Weidenbacher PA , Tang S , Wu W , Pak JE and Kim PS (2024) Efficient evolution of human antibodies from general protein language models. Nat Biotechnol 42, 275–283. 37095349 10.1038/s41587-023-01763-2 PMC10869273

[58] Dauparas J , Anishchenko I , Bennett N , Bai H , Ragotte RJ , Milles LF , Wicky BIM , Courbet A , de Haas RJ , Bethel N *et al*. (2022) Robust deep learning‐based protein sequence design using ProteinMPNN. Science 378, 49–56. 36108050 10.1126/science.add2187 PMC9997061

[59] Bennett NR , Coventry B , Goreshnik I , Huang B , Allen A , Vafeados D , Peng YP , Dauparas J , Baek M , Stewart L *et al*. (2023) Improving de novo protein binder design with deep learning. Nat Commun 14, 2625. 37149653 10.1038/s41467-023-38328-5 PMC10163288

[60] Bertoline LMF , Lima AN , Krieger JE and Teixeira SK (2023) Before and after AlphaFold2: an overview of protein structure prediction. Front Bioinform 3, 1120370. 36926275 10.3389/fbinf.2023.1120370 PMC10011655

[61] Ferruz N , Heinzinger M , Akdel M , Goncearenco A , Naef L and Dallago C (2023) From sequence to function through structure: deep learning for protein design. Comput Struct Biotechnol J 21, 238–250. 36544476 10.1016/j.csbj.2022.11.014 PMC9755234

[62] Krapp LF , Abriata LA , Cortes Rodriguez F and Dal Peraro M (2023) PeSTo: parameter‐free geometric deep learning for accurate prediction of protein binding interfaces. Nat Commun 14, 2175. 37072397 10.1038/s41467-023-37701-8 PMC10113261

[63] Evans R , O'Neill M , Pritzel A , Antropova N , Senior A , Green T , Žídek A , Bates R , Blackwell S , Yim J *et al*. (2022) Protein complex prediction with AlphaFold‐multimer. bioRxiv. doi: 10.1101/2021.10.04.463034

[64] Yin R , Feng BY , Varshney A and Pierce BG (2022) Benchmarking AlphaFold for protein complex modeling reveals accuracy determinants. Protein Sci 31, e4379. 35900023 10.1002/pro.4379 PMC9278006

[65] Abramson J , Adler J , Dunger J , Evans R , Green T , Pritzel A , Ronneberger O , Willmore L , Ballard AJ , Bambrick J *et al*. (2024) Accurate structure prediction of biomolecular interactions with AlphaFold 3. Nature 630, 493–500. 38718835 10.1038/s41586-024-07487-w PMC11168924

[66] Baek M , DiMaio F , Anishchenko I , Dauparas J , Ovchinnikov S , Lee GR , Wang J , Cong Q , Kinch LN , Schaeffer RD *et al*. (2021) Accurate prediction of protein structures and interactions using a three‐track neural network. Science 373, 871–876. 34282049 10.1126/science.abj8754 PMC7612213

[67] Zhang Y and Skolnick J (2005) TM‐align: a protein structure alignment algorithm based on the TM‐score. Nucleic Acids Res 33, 2302–2309. 15849316 10.1093/nar/gki524 PMC1084323

[68] Lin Z , Akin H , Rao R , Hie B , Zhu Z , Lu W , Smetanin N , Verkuil R , Kabeli O , Shmueli Y *et al*. (2023) Evolutionary‐scale prediction of atomic‐level protein structure with a language model. Science 379, 1123–1130. 36927031 10.1126/science.ade2574

[69] Metropolis N , Rosenbluth AW , Rosenbluth MN , Teller AH and Teller E (1953) Equation of state calculations by fast computing machines. J Chem Phys 21, 1087. 10.1063/5.0309018 41342507

[70] Bender BJ , Cisneros A , Duran AM , Finn JA , Fu D , Lokits AD , Mueller BK , Sangha AK , Sauer MF , Sevy AM *et al*. (2016) Protocols for molecular modeling with Rosetta3 and RosettaScripts. Biochemistry 55, 4748–4763. 27490953 10.1021/acs.biochem.6b00444 PMC5007558

[71] Bellissent‐Funel M‐C , Hassanali A , Havenith M , Henchman R , Pohl P , Sterpone F , van der Spoel D , Xu Y and Garcia AE (2016) Water determines the structure and dynamics of proteins. Chem Rev 116, 7673–7697. 27186992 10.1021/acs.chemrev.5b00664 PMC7116073

[72] Guo Z and Yamaguchi R (2022) Machine learning methods for protein–protein binding affinity prediction in protein design. Front Bioinform 2, 1065703. 36591334 10.3389/fbinf.2022.1065703 PMC9800603

[73] Wan S , Bhati AP , Zasada SJ and Coveney PV (2020) Rapid, accurate, precise and reproducible ligand–protein binding free energy prediction. Interface Focus 10, 20200007. 33178418 10.1098/rsfs.2020.0007 PMC7653346

[74] Martin HG , Radivojevic T , Zucker J , Bouchard K , Sustarich J , Peisert S , Arnold D , Hillson N , Babnigg G , Marti JM *et al*. (2023) Perspectives for self‐driving labs in synthetic biology. Curr Opin Biotechnol 79, 102881. 36603501 10.1016/j.copbio.2022.102881

[75] Rapp JT , Bremer BJ and Romero PA (2024) Self‐driving laboratories to autonomously navigate the protein fitness landscape. Nat Chem Eng 1, 97–107. 38468718 10.1038/s44286-023-00002-4 PMC10926838

